# Postpartum depression in Northeastern China: a cross-sectional study 6 weeks after giving birth

**DOI:** 10.3389/fpubh.2025.1570654

**Published:** 2025-05-21

**Authors:** XuDong Huang, LiFeng Zhang, ChenYang Zhang, Jing Li, ChenYang Li

**Affiliations:** ^1^Department of Science and Education, Shenyang Maternity and Child Health Hospital, Shenyang, China; ^2^Department of Maternal, Child and Adolescent Health, Shenyang Medical College, School of Public Health, Shenyang, China

**Keywords:** postpartum depression, prevalence, risk factors, cross-sectional study, depression

## Abstract

**Introduction:**

Postpartum depression (PPD) is a prevalent mental health issue that poses significant challenges to maternal wellbeing and infant development. We aimed to determine the prevalence of PPD and to investigate its associated determinants and predictors in in Shenyang, China.

**Methods:**

This cross-sectional study, conducted between January and December 2021, included 1,065 postpartum women in Shenyang, China, at 6 weeks postpartum. PPD was screened using the Edinburgh Postnatal Depression Scale (EPDS, score ≥ 9). Key risk factors were identified through machine learning techniques, including LASSO regression and the Boruta algorithm, and their associations were evaluated using logistic regression. Non-linear relationships for continuous variables were examined using restricted cubic splines and threshold effect analysis. Feature importance was ranked via a random forest model based on the change in ROC-AUC after predictor removal. Statistical significance was defined as *p* < 0.05.

**Results:**

A total of 1,065 postpartum mothers were included in this study, of whom 23.57% were identified as having postpartum depressive symptoms. Significant risk factors included prenatal anxiety (OR = 7.16, 95% CI: 4.67–11.11), poor sleep quality (OR = 5.30, 95% CI: 3.45–8.20), poor marital relationships (OR = 4.41, 95% CI: 2.47–7.99), poor in-law relationships (OR = 4.89, 95% CI: 3.11–7.74), unplanned pregnancy (OR = 2.92, 95% CI: 2.01–4.27), and lack of prenatal education (OR = 1.7, 95% CI: 1.15–2.52). A non-linear relationship was observed with gestational weight gain: gains <11 kg were associated with reduced risk (OR = 0.91, 95% CI: 0.83–0.99), whereas gains >11 kg increased risk (OR = 1.06, 95% CI: 1.02–1.10). Other factors included smoking history (OR = 1.8, 95% CI: 1.08–2.98) and expected fetal sex (OR = 3.39, 95% CI: 2.02–5.70).

**Conclusion:**

PPD affects a significant proportion of postpartum women in Shenyang, China. Routine screening and targeted interventions are essential to identify and support high-risk individuals with PPD.

## 1 Introduction

Postpartum depression (PPD) is a prevalent mental health condition following childbirth, characterized by persistent depressive symptoms and emotional distress (1, 2). Epidemiological studies reveal significant global variability in PPD prevalence, ranging from 5.0% to 26.32% (3). In China, the prevalence is estimated at 21.4% (95% CI: 15.2%−27.6%) and is on the rise (3, 4). PPD profoundly impacts maternal physical and mental health, significantly impairing quality of life (1, 5). It also disrupts the mother-infant relationship, potentially hindering newborns' cognitive, emotional, and behavioral development, with long-term adverse outcomes (2, 6–9). Given these severe consequences, early identification of high-risk postpartum women and effective interventions are essential to mitigating PPD prevalence and promoting maternal and infant health.

PPD arises from a complex interplay of biological, environmental, and psychosocial factors (1). Studies have identified various potential risk factors, including prenatal anxiety, low educational attainment, adverse marital relationships, inadequate social support, delivery method, and obesity (3, 4, 9). However, the underlying causes of PPD remain ambiguous, with findings often varying across studies (2, 6). Current research predominantly emphasizes psychosocial factors, whereas postpartum gynecological examinations, such as pelvic floor muscle assessments, remain underexplored. Additionally, cultural, policy, and welfare system differences contribute to regional variability in PPD risk factors. In China, most studies have concentrated on southern regions and coastal cities, leaving northeastern areas, such as Shenyang, relatively underrepresented. As a city undergoing industrial restructuring, Shenyang may face economic challenges distinct from those in more economically developed regions, potentially impacting familial financial burdens—a recognized risk factor for PPD (10). Additionally, with an aging population exceeding the national average and a prevalent “grandparenting” model, Shenyang may experience more pronounced intergenerational parenting conflicts compared to regions where nuclear family structures are more common (11). By examining PPD in this context, our study seeks to offer insights specific to Shenyang and inform targeted interventions and healthcare policies.

Given the profound impact of PPD on maternal and infant health, coupled with regional disparities in its prevalence and associated risk factors, we conducted a cross-sectional study to assess PPD among women in Shenyang, China, at 6 weeks postpartum. This study aims to determine the prevalence of PPD and identify its related risk factors, integrating both common sociodemographic characteristics and data from routine clinical evaluations performed at 42 days postpartum. By analyzing these factors, our research seeks to provide robust scientific evidence to inform screening protocols, prevention strategies, and policy development for PPD management.

## 2 Methods

### 2.1 Study design and participants

This cross-sectional study was conducted at a tertiary maternal and child health hospital in Shenyang, China, including postpartum women who attended a 42-day follow-up visit between January and December 2021. The inclusion criteria were: (1) women attending follow-up visits at 6 weeks postpartum; (2) women with available PPD assessment results; and (3) women who voluntarily consented to participate in the study. Exclusion criteria were: (1) women with severe organic diseases, including cardiovascular, hepatic, renal, or pulmonary disorders; (2) women with incomplete demographic data, making follow-up contact impossible; and (3) women who declined participation.This study was approved by the Hospital Ethics Committee (Approval Number: 202301701). Each participant was informed that the data collected from her might be used for research and publication purposes, and written consent was obtained from each woman.

### 2.2 Sample size and sampling

The sample size calculation was based on the standard formula for prevalence studies:


$$\begin{array}{l} n=\frac{Z^{2}\cdot P\cdot \left(1-P\right)}{d^{2}} \end{array}$$

where n is the required sample size, Z = 1.96 corresponds to a 95% confidence level, *P* = 0.214 represents the expected prevalence of postpartum depression (3) (based on prior literature reporting a 21.4% prevalence rate among women in China at 6 weeks postpartum), and d = 0.032 is the margin of error (set at 15% of the expected prevalence, i.e., 0.15 × 0.214). This calculation yielded a minimum sample size of 627 participants. A convenience sampling technique was used to recruit postpartum women who attended the 42-day check-up between January and December 2021. Women who met the inclusion criteria and voluntarily consented to participate were enrolled. This approach was chosen for its feasibility and efficiency in participant recruitment. In this study, we surveyed 1,065 postpartum women, exceeding the minimum requirement and ensuring sufficient statistical power.

### 2.3 Data collection and study variables

#### 2.3.1 Data collection methods

Based on a literature review and expert consultations (10, 12), we designed a structured questionnaire in Chinese to systematically collect relevant information from participants. Data collection was conducted as follows: we reviewed standardized routine examination records and past medical histories completed by all participants at 42 days postpartum to obtain clinical data, including pregnancy, delivery information, and postpartum health status. All routine examinations were conducted by professional healthcare personnel to ensure the reliability and consistency of the clinical data. Data on psychosocial factors, encompassing basic demographic information and psychosocial elements, were collected through structured telephone interviews conducted by trained researchers.

#### 2.3.2 PPD screening criteria

PPD was assessed using the Chinese version of the Edinburgh Postnatal Depression Scale (EPDS), a widely used self-report screening tool for postpartum depression. The EPDS consists of 10 items scored from 0 (never) to 3 (almost always) based on symptom frequency, with a total score range of 0 to 30. Higher scores reflect more severe depressive symptoms. The Chinese version of the EPDS has demonstrated good validity and reliability, with a sensitivity of 0.82 and a specificity of 0.86, comparable to the diagnostic accuracy of the original scale (9). In this study, an EPDS score of ≥9 was used as the threshold for PPD screening, consistent with recommendations from prior validation studies.

#### 2.3.3 Demographics and basic information

Age, educational level, Body Mass Index (BMI) measured at the 42-day postpartum check-up, family economic status, smoking history, alcohol consumption history, and pre-pregnancy menstrual cycle abnormalities (defined as cycles shorter than 21 days or longer than 35 days).

#### 2.3.4 Pregnancy and delivery information

Nullipara, adverse obstetric history (including spontaneous abortion, induced abortion, termination of pregnancy, and ectopic pregnancy), gestational weight gain (kg), pregnancy related conditions (thyroid disorders, gestational hypertension, gestational diabetes), cesarean delivery, painless delivery, episiotomy, perineal laceration, instrumental delivery, manual placenta removal, number of fetuses delivered, premature birth, abnormal fetal weight (birth weight < 2,500 g or ≥4,000 g), and adequacy of breast milk.

#### 2.3.5 Postpartum health conditions

The assessment included postpartum pain (any pain, of any location or duration, self-reported by the mother during the postpartum period), as well as urinary dysfunction and bowel dysfunction, both also self-reported by the mothers. In addition, several postpartum conditions were assessed by doctors or nurses during the 42-day postpartum check-up, including vaginal bleeding/discharge, findings from vaginal, cervical, uterine, and adnexal examinations, hemorrhoids, abdominal scars, pelvic floor muscle strength (including both type I and type II muscle fibers), pelvic floor muscle endurance, pelvic floor muscle tenderness, and pelvic dynamic pressure (cmH_2_O). Pelvic floor muscle strength, encompassing both type I and type II muscle fibers, was assessed through vaginal palpation using the Oxford grading system, which ranges from 0 to 5 (13). A grade of 0 indicates no muscle contraction, while a grade of 5 signifies the strongest contraction. Pelvic floor muscle endurance is primarily evaluated based on the duration and repetition of muscle contractions, with a grading scale from 0 to 5 s. For detailed information on the relevant examinations, please refer to the supplementary document.

#### 2.3.6 Psychological and social factors

Perinatal sleep quality, fetal sex preference, attendance at prenatal education classes, prenatal anxiety, satisfaction with postpartum confinement, marital relationship, and in-law relationship were assessed based on self-reports from postpartum women.

### 2.4 Data analysis

Data entry was conducted using EpiData 3.1 software, employing a double-entry verification process to ensure accuracy. Statistical analyses were performed with DecisionLinnc 1.1.1.9 software (https://www.statsape.com/). For variables with missing data rates below 20%, imputation was applied using the k-nearest neighbors algorithm for continuous variables and multiple imputation for categorical variables; variables with missing data exceeding 20% were excluded to maintain analytical integrity. The Kolmogorov-Smirnov test confirmed the non-normal distribution of continuous variables, which were then summarized as medians with interquartile ranges (IQRs) and compared using the Mann-Whitney U test. Categorical variables were expressed as percentages and analyzed with Pearson's chi-squared test.

To address the inclusion of numerous independent variables in this study, we implemented a hybrid approach combining machine learning-based variable selection and multivariable logistic regression to efficiently identify key predictors of PPD while minimizing multicollinearity among variables. Initially, we employed LASSO regression for variable selection, using regularization to reduce the number of predictors and highlight the most relevant factors. The optimal penalty parameter (λ) was set at lambda.min, determined by minimizing cross-validation error. Following this, the Boruta algorithm was applied to the features selected by LASSO for further refinement. Boruta operates within a tree-based framework to assess variable importance, effectively identifying significant predictors while managing non-linear relationships and high-dimensional data. The variables selected through this process were then incorporated into a multivariable logistic regression model to quantify their associations with PPD. To ensure the robustness of the model, we conducted a variance inflation factor (VIF) diagnosis to assess multicollinearity among the independent variables. Non-linear relationships of significant continuous variables were explored using restricted cubic splines (RCS) and threshold effect analysis, enabling the identification of inflection points and the calculation of odds ratios (ORs) and confidence intervals (CIs) at various thresholds. Additionally, feature importance was evaluated using a random forest model, with predictors ranked based on changes in the area under the receiver operating characteristic curve (ROC-AUC) following their sequential removal. Statistical significance was set at a threshold of *p* < 0.05.

## 3 Results

### 3.1 Prevalence and demographic associations of PPD

This study included 1,065 postpartum women, among whom 251 had EPDS scores ≥9, yielding a PPD detection rate of 23.57%. Participants' ages ranged from 18 to 43 years, with a median age of 29 years. Table 1 compares the basic demographic characteristics of postpartum women with and without PPD. Univariate analysis of maternal demographics showed significant associations between PPD and age (*P* = 0.020), education level (*P* = 0.022), economic status (*P* = 0.001), smoking history (*P* < 0.001), and pre-pregnancy menstrual cycle abnormalities (*P* = 0.013).

**Table 1:** Comparison of baseline characteristics between patients with PPD and non-PPD patients.

**Variables**	**Total (*N* = 1,065)**	**Non-PPD (*N* = 814)**	**PPD (*N* = 251)**	***P*-value**
Age (years), M (Q1, Q3)	29 (27–32)	29 (27–32)	29 (26–31)	0.020
**Education (%)**				
Below high school	93 (8.73)	65 (7.99)	28 (11.16)	0.022
Below bachelor's degree	536 (50.33)	398 (48.89)	138 (54.98)	
Bachelor's degree or above	436 (40.94)	351 (43.12)	85 (33.86)	
**Economic level (%)**				
Poor	432 (40.56)	316 (38.82)	116 (46.22)	0.001
Moderate	516 (48.45)	419 (51.47)	97 (38.65)	
Good	117 (10.99)	79 (9.71)	38 (15.14)	
**Smoking history (%)**				
Yes	125 (11.74)	79 (9.71)	46 (18.33)	< 0.001
No	940 (88.26)	735 (90.29)	205 (81.67)	
**Alcohol consumption history (%)**				
Yes	78 (7.32)	58 (7.13)	20 (7.97)	0.757
No	987 (92.68)	756 (92.87)	231 (92.03)	
Body Mass Index (kg/m^2^), M (Q1, Q3)	24.038 (22.231–26.346)	24.093 (22.309–26.444)	23.855 (21.859–26.195)	0.384
**Pre-pregnancy menstrual cycle abnormalities (%)**				
Yes	132 (12.39)	89 (10.93)	43 (17.13)	0.013
No	933 (87.61)	725 (89.07)	208 (82.87)	

PPD, postpartum depression; M, Median; Q1, 1st Quartile; Q3, 3st Quartile.

### 3.2 Pregnancy and delivery factors associated with PPD

The univariate analysis of pregnancy and delivery information revealed that only weight gain during pregnancy (*P* = 0.011) and adequate breast milk supply (*P* = 0.008) were significantly associated with PPD. Other factors, including nullipara, adverse obstetric history, thyroid abnormalities, pregnancy-induced hypertension, gestational diabetes, pregnancy related conditions, cesarean section, painless delivery, episiotomy, perineal laceration, manual removal of placenta, forceps delivery, preterm birth, twin birth, and abnormal fetal weight, showed no significant associations with PPD. The results are presented in Table 2.

**Table 2:** Univariate analysis of pregnancy and delivery factors associated with PPD.

**Variables**	**Total (*N* = 1,065)**	**Non-PPD (*N* = 814)**	**PPD (*N* = 251)**	***P*-value**
**Nullipara (%)**				
Yes	866 (81.31)	657 (80.71)	209 (83.27)	0.415
No	199 (18.69)	157 (19.29)	42 (16.73)	
**Adverse obstetric history (%)**				
Yes	337 (31.64)	246 (30.22)	91 (36.25)	0.086
No	728 (68.36)	568 (69.78)	160 (63.75)	
**Thyroid abnormalities during pregnancy (%)**				
Yes	44 (4.13)	35 (4.30)	9 (3.59)	0.752
No	1,021 (95.87)	779 (95.70)	242 (96.41)	
**Pregnancy induced hypertension (%)**				
Yes	29 (2.72)	23 (2.83)	6 (2.39)	0.882
No	1,036 (97.28)	791 (97.17)	245 (97.61)	
**Gestational diabetes (%)**				
Yes	169 (15.87)	131 (16.09)	38 (15.14)	0.793
No	896 (84.13)	683 (83.91)	213 (84.86)	
**Pregnancy related conditions (%)**				
Yes	242 (22.72)	189 (23.22)	53 (21.12)	0.543
No	823 (77.28)	625 (76.78)	198 (78.88)	
Gestational weight gain (Kg), M (Q1, Q3)	15 (11–20)	15 (11–19)	15 (12–20)	0.011
**Cesarean section (%)**				
Yes	448 (42.07)	336 (41.28)	112 (44.62)	0.387
No	617 (57.93)	478 (58.72)	139 (55.38)	
**Painless delivery (%)**				
Yes	551 (51.74)	421 (51.72)	130 (51.79)	1.000
No	514 (48.26)	393 (48.28)	121 (48.21)	
**Episiotomy (%)**				
Yes	192 (18.03)	145 (17.81)	47 (18.73)	0.814
No	873 (81.97)	669 (82.19)	204 (81.27)	
**Perineal laceration (%)**				
Yes	453 (42.54)	345 (42.38)	108 (43.03)	0.914
No	612 (57.46)	469 (57.62)	143 (56.97)	
**Manual removal of placenta (%)**				
Yes	158 (14.84)	118 (14.50)	40 (15.94)	0.646
No	907 (85.16)	696 (85.50)	211 (84.06)	
**Forceps delivery (%)**				
Yes	32 (3.00)	23 (2.83)	9 (3.59)	0.685
No	1,033 (97.00)	791 (97.17)	242 (96.41)	
**Preterm birth (%)**				
Yes	73 (6.85)	51 (6.27)	22 (8.76)	0.220
No	992 (93.15)	763 (93.73)	229 (91.24)	
**Twin birth (%)**				
Yes	10 (0.94)	7 (0.86)	3 (1.20)	0.915
**Abnormal fetal weight (%)**				
Yes	409 (38.40)	304 (37.35)	105 (41.83)	0.229
No	656 (61.60)	510 (62.65)	146 (58.17)	
**Adequate breast milk (%)**				
Yes	684 (64.23)	541 (66.46)	143 (56.97)	0.008
No	381 (35.77)	273 (33.54)	108 (43.03)	

PPD, postpartum depression; M, Median; Q1, 1st Quartile; Q3, 3st Quartile; Pregnancy related conditions, including thyroid disorders, gestational hypertension, and gestational diabetes; adverse obstetric history, including spontaneous abortion, induced abortion, termination of pregnancy, and ectopic pregnancy.

### 3.3 Postpartum health conditions associated with PPD

In this study, univariate analysis revealed that PPD was significantly associated with postpartum pain (*P* = 0.004), urinary dysfunction (*P* = 0.019), and pubic symphysis pain (*P* = 0.044). However, there were no significant associations between PPD and other postpartum health conditions such as diastasis recti, pelvic pressure, bowel dysfunction, vaginal bleeding, abdominal scars, vulva and vagina examination results, cervix, uterus, and adnexa examination results, hemorrhoids, pelvic floor tenderness, and the distribution of pelvic floor muscle strength and endurance. The results are presented in Table 3.

**Table 3:** Univariate analysis of postpartum health conditions related to PPD.

**Variables**	**Total (*N* = 1,065)**	**Non-PPD (*N* = 814)**	**PPD (*N* = 251)**	***P*-value**
Diastasis Recti (cm), M (Q1, Q3)	2 (1.5–2.5)	2 (1.5–2.5)	2 (1.5–2.5)	0.195
Pelvic pressure (cmH_2_O), M (Q1, Q3)	63 (57–72)	63 (57–71)	63.2 (58–75)	0.552
**Postpartum pain (%)**				
Yes	222 (20.85)	153 (18.80)	69 (27.49)	0.004
No	843 (79.15)	661 (81.20)	182 (72.51)	
**Urinary dysfunction (%)**				
Yes	157 (14.74)	108 (13.27)	49 (19.52)	0.019
No	908 (85.26)	706 (86.73)	202 (80.48)	
**Bowel dysfunction (%)**				
Yes	166 (15.59)	122 (14.99)	44 (17.53)	0.384
No	899 (84.41)	692 (85.01)	207 (82.47)	
**Vaginal bleeding/discharge (%)**				
Yes	216 (20.28)	162 (19.90)	54 (21.51)	0.642
No	849 (79.72)	652 (80.10)	197 (78.49)	
**Abdominal scar (%)**				
Yes	422 (39.62)	312 (38.33)	110 (43.82)	0.138
No	643 (60.38)	502 (61.67)	141 (56.18)	
**Pubic symphysis pain (%)**				
Yes	265 (24.88)	190 (23.34)	75 (29.88)	0.044
No	800 (75.12)	624 (76.66)	176 (70.12)	
**Vulva examination (%)**				
Normal	624 (58.59)	476 (58.48)	148 (58.96)	0.949
Abnormal	441 (41.41)	338 (41.52)	103 (41.04)	
**Vagina examination (%)**				
Normal	835 (78.40)	638 (78.38)	197 (78.49)	1.000
Abnormal	230 (21.60)	176 (21.62)	54 (21.51)	
**Cervix examination (%)**				
Normal	916 (86.01)	704 (86.49)	212 (84.46)	0.481
Abnormal	149 (13.99)	110 (13.51)	39 (15.54)	
**Uterus examination (%)**				
Normal	1,037 (97.37)	792 (97.30)	245 (97.61)	0.964
Abnormal	28 (2.63)	22 (2.70)	6 (2.39)	
**Adnexa examination (%)**				
Normal	1,033 (97.00)	789 (96.93)	244 (97.21)	0.986
Abnormal	32 (3.00)	25 (3.07)	7 (2.79)	
**Hemorrhoids (%)**				
Yes	453 (42.54)	341 (41.89)	112 (44.62)	0.489
No	612 (57.46)	473 (58.11)	139 (55.38)	
**Pelvic Floor Tenderness (%)**				
Yes	89 (8.36)	67 (8.23)	22 (8.76)	0.891
No	976 (91.64)	747 (91.77)	229 (91.24)	
**Grade distribution of pelvic floor muscle strength (%)**				
0	22 (2.07)	19 (2.33)	3 (1.20)	0.180
1	381 (35.77)	305 (37.47)	76 (30.28)	
2	355 (33.33)	258 (31.70)	97 (38.65)	
3	206 (19.34)	155 (19.04)	51 (20.32)	
4	78 (7.32)	58 (7.13)	20 (7.97)	
5	23 (2.16)	19 (2.33)	4 (1.59)	
**Pelvic floor muscle endurance (%)**				
0s	78 (7.32)	65 (7.99)	13 (5.18)	0.363
1s	498 (46.76)	387 (47.54)	111 (44.22)	
2s	285 (26.76)	215 (26.41)	70 (27.89)	
3s	135 (12.68)	97 (11.92)	38 (15.14)	
4s	50 (4.69)	35 (4.30)	15 (5.98)	
5s	19 (1.78)	15 (1.84)	4 (1.59)	
**Type I pelvic floor muscles (%)**				
0	315 (29.58)	244 (29.98)	71 (28.29)	0.956
1	273 (25.63)	209 (25.68)	64 (25.50)	
2	131 (12.30)	100 (12.29)	31 (12.35)	
3	80 (7.51)	62 (7.62)	18 (7.17)	
4	34 (3.19)	27 (3.32)	7 (2.79)	
5	232 (21.78)	172 (21.13)	60 (23.90)	
**Type II pelvic floor muscles (%)**				
0	212 (19.91)	160 (19.66)	52 (20.72)	0.914
1	144 (13.52)	113 (13.88)	31 (12.35)	
2	123 (11.55)	98 (12.04)	25 (9.96)	
3	99 (9.30)	75 (9.21)	24 (9.56)	
4	74 (6.95)	57 (7.00)	17 (6.77)	
5	413 (38.78)	311 (38.21)	102 (40.64)	

PPD, postpartum depression; M, Median; Q1, 1st Quartile; Q3, 3st Quartile.

### 3.4 Psychosocial factors associated with PPD

In this study, univariate analysis of psychosocial factors revealed significant associations with PPD. Specifically, unexpected fetal sex (*P* < 0.001), unplanned pregnancy (*P* < 0.001), lack of prenatal education class attendance (*P* = 0.041), poor perinatal sleep quality (*P* < 0.001), prenatal anxiety (*P* < 0.001), dissatisfaction with postpartum confinement (*P* = 0.002), poor marital relationship (*P* < 0.001), and poor mother-in-law and daughter-in-law relationship (*P* < 0.001) were all significantly associated with higher rates of PPD. The results are presented in Table 4.

**Table 4:** Univariate analysis of psychosocial factors related to PPD.

**Variables**	**Total (*N* = 1,065)**	**Non-PPD (*N* = 814)**	**PPD (*N* = 251)**	***P*-value**
**Fetal sex (%)**				
Expected	778 (73.05)	618 (75.92)	160 (63.75)	< 0.001
Unexpected	287 (26.95)	196 (24.08)	91 (36.25)	
**Planned pregnancy (%)**				
Yes	745 (69.95)	598 (73.46)	147 (58.57)	< 0.001
No	320 (30.05)	216 (26.54)	104 (41.43)	
**Prenatal education class (%)**				
Yes	404 (37.93)	322 (39.68)	81 (32.27)	0.041
No	661 (62.07)	491 (60.32)	170 (67.73)	
**Perinatal sleep quality (%)**				
Good	626 (58.78)	510 (62.65)	116 (46.22)	< 0.001
Average	268 (25.16)	223 (27.40)	45 (17.93)	
Poor	171 (16.06)	81 (9.95)	90 (35.86)	
**Prenatal anxiety (%)**				
Yes	217 (20.38)	116 (14.25)	101 (40.24)	< 0.001
No	848 (79.62)	698 (85.75)	150 (59.76)	
**Satisfaction with postpartum confinement (%)**				
Satisfied	762 (71.55)	602 (73.96)	160 (63.75)	0.002
Unsatisfied	303 (28.45)	212 (26.04)	91 (36.25)	
**Marital relationship (%)**				
Good	955 (89.67)	759 (93.24)	196 (78.09)	< 0.001
Poor	110 (10.33)	55 (6.76)	55 (21.91)	
**Mother-in-law and daughter-in-law relationship (%)**				
Good	879 (82.54)	708 (86.98)	171 (68.13)	< 0.001
Poor	186 (17.46)	106 (13.02)	80 (31.87)	

PPD, postpartum depression.

### 3.5 Feature selection with LASSO and Boruta

**Figure 1:**
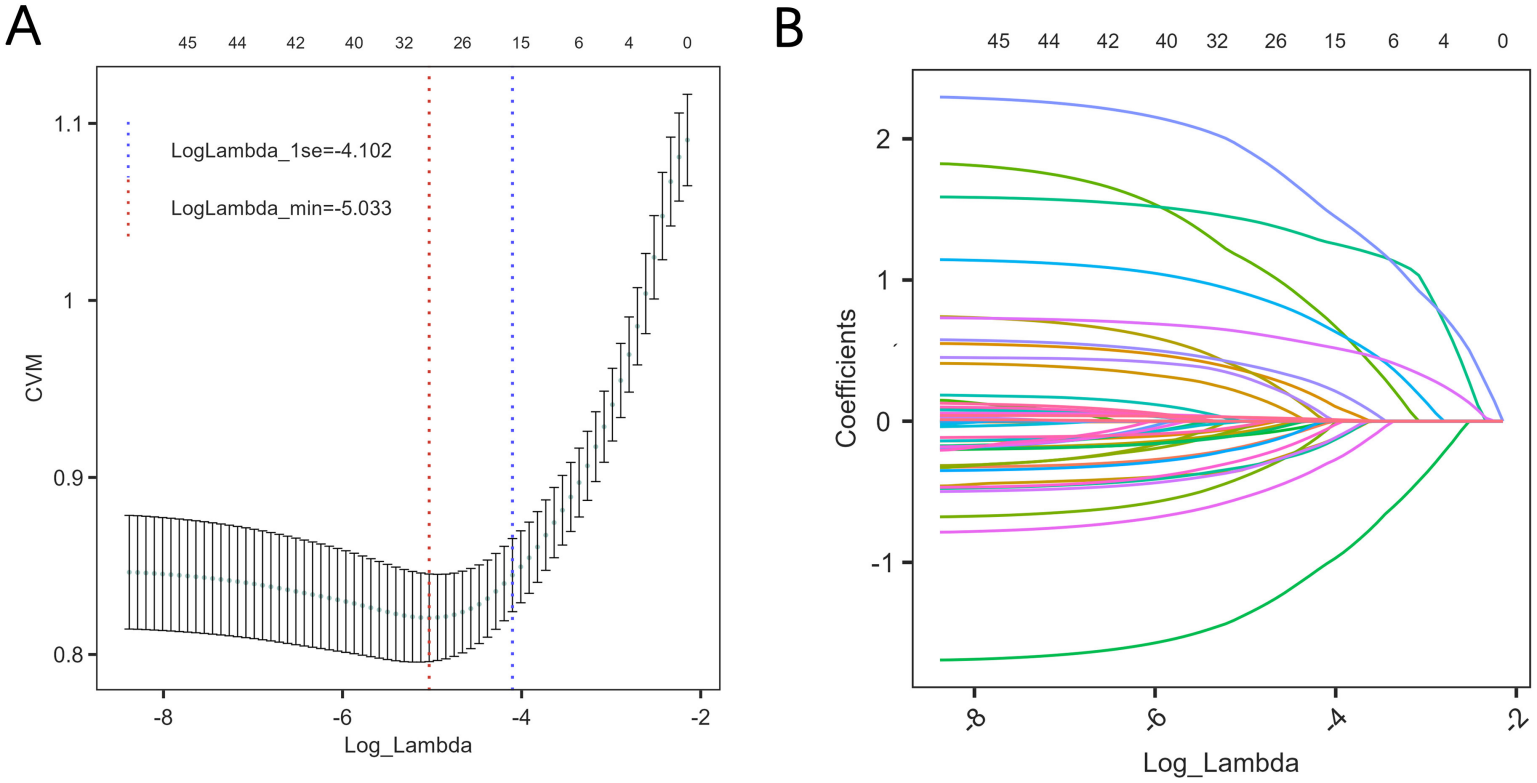
Variable selection using Lasso regression. **(A)** Profiles of Lasso coefficients for each predictor as a function of the regularization parameter (log Lambda). **(B)** Selection of the optimal value of lambda via ten-fold cross-validation. The cross-validated error (CVM) is plotted against log(Lambda); the vertical dashed lines indicate the values of lambda corresponding to the minimum error (lambda.min) and the most parsimonious model within one standard error of the minimum (lambda.1se).

**Figure 2:**
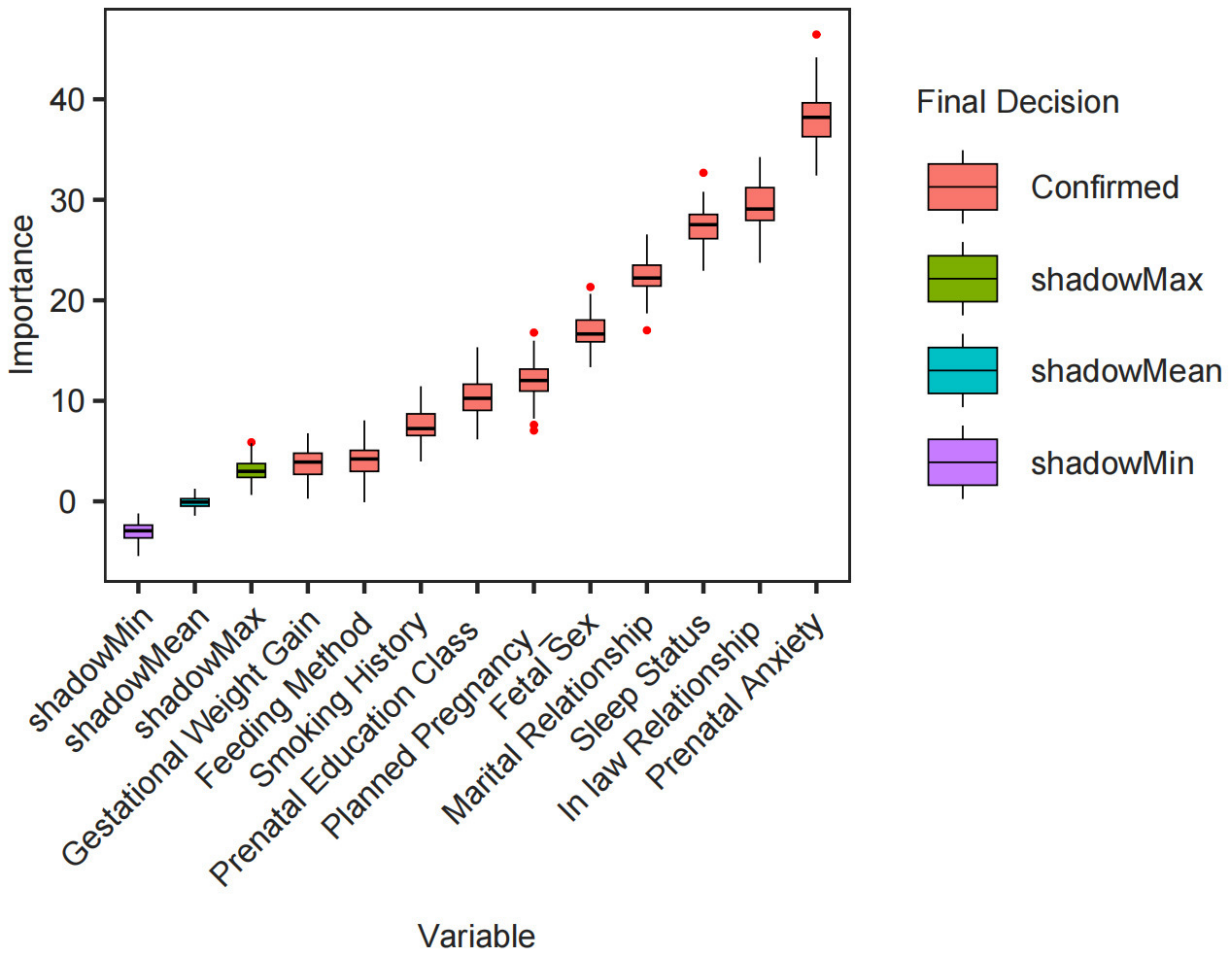
Variable selection using the Boruta algorithm. Box plots show the importance scores of each variable as calculated by the Boruta feature selection algorithm. “Confirmed” refers to variables with statistically significant importance (red), while “shadowMax,” “shadowMean,” and “shadowMin” represent the maximum, average, and minimum importance scores among shadow (randomized) variables (green, blue, and purple, respectively). Variables with confirmed importance scores exceeding the shadow benchmarks are considered truly informative for the model.

Initially, LASSO regression was employed for a preliminary screening of all variables, identifying 18 variables associated with PPD at lambda.min = −4.102 (Figure 1). Subsequently, the Boruta algorithm was applied to refine the selection, resulting in 10 key features: smoking history, gestational weight gain, feeding method, fetal sex, planned pregnancy, prenatal education class, prenatal anxiety, sleep quality, marital relationship, and the mother-in-law and daughter-in-law relationship (Figure 2). These features were incorporated into the final logistic regression analysis.

### 3.6 Multivariate analysis: predictors of PPD

A multivariate logistic regression analysis was conducted with PPD occurrence as the dependent variable and the 10 variables identified by the Boruta algorithm as independent variables. The analysis indicated that smoking history, gestational weight gain, expected fetal sex, unplanned pregnancy, lack of attendance at prenatal education classes, prenatal anxiety, poor sleep quality, poor marital relationship, and poor in-law relationship were significant factors influencing PPD among postpartum women at 6 weeks (*P* < 0.05), as detailed in Table 5. Additionally, a collinearity diagnosis was performed for the independent variables, and all VIF were found to be < 5, as shown in Supplementary Table 1.

**Table 5:** Multivariate analysis of risk factor for postpartum depressive symptoms.

**Variables**	**β**	**S.E**	**Z**	***P***	**OR (95%CI)**
Intercept	−2.62	0.35	−7.53	< 0.001	0.07 (0.04, 0.14)
**Smoking history**					
No					1.00 (Reference)
Yes	0.59	0.26	2.29	0.02	1.8 (1.08, 2.98)
Gestational weight gain	0.03	0.01	2.17	0.03	1.03 (1.0, 1.06)
**Fetal sex**					
Unexpected					1.00 (Reference)
Expected	1.22	0.26	4.62	< 0.001	3.39 (2.02, 5.7)
**Planned pregnancy**					
Yes					1.00 (Reference)
No	1.07	0.19	5.61	< 0.001	2.92 (2.01, 4.27)
**Prenatal education class**					
Yes					1.00 (Reference)
No	0.53	0.2	2.65	0.01	1.7 (1.15, 2.52)
**Prenatal anxiety**					
No					1.00 (Reference)
Yes	1.97	0.22	8.91	< 0.001	7.16 (4.67, 11.11)
**Perinatal sleep quality**					
Good					1.00 (Reference)
Poor	1.67	0.22	7.55	< 0.001	5.3 (3.45, 8.2)
**Marital relationship**					
Good					1.00 (Reference)
Poor	1.48	0.3	4.96	< 0.001	4.41 (2.47, 7.99)
**Mother-in-law and daughter-in-law relationship**					
Good					1.00 (Reference)
Poor	1.59	0.23	6.83	< 0.001	4.89 (3.11, 7.74)

### 3.7 Dose-response relationship between gestational weight gain and PPD

**Figure 3:**
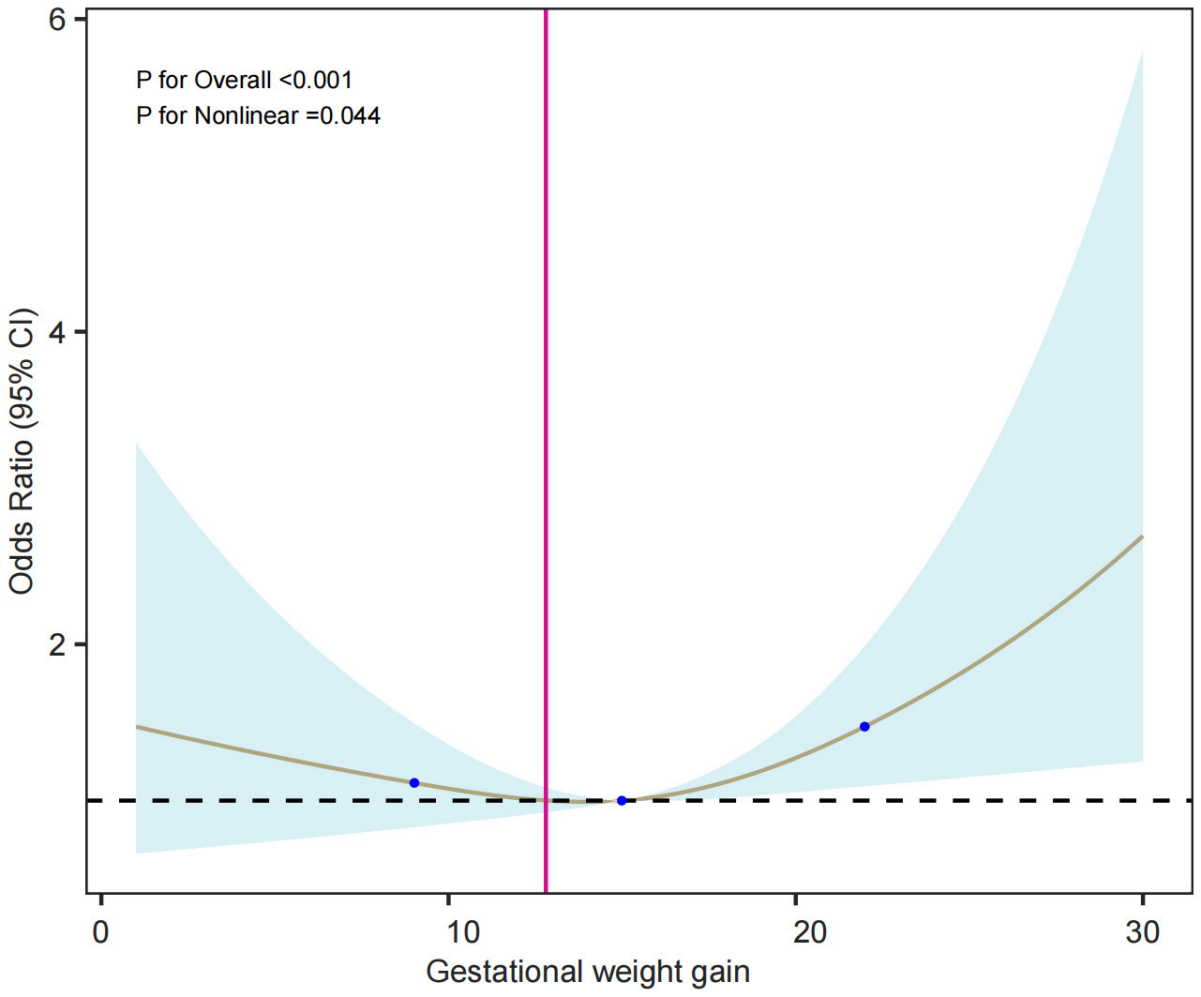
Restricted cubic spline (RCS) model of gestational weight gain and risk of PPD. Adjusted for smoking, fetal sex, planned pregnancy, prenatal education, prenatal anxiety, perinatal sleep quality, marital relationship, and relationship with in-laws. The solid line shows the estimated odds ratio, the blue shaded area the 95% confidence interval, and blue dots indicate knot locations.

The relationship between gestational weight gain and PPD was explored using RCS and threshold effect analysis. Multivariable-adjusted RCS analysis identified a significant non-linear relationship between gestational weight gain and PPD (*P* for non-linearity = 0.044). This relationship is illustrated in Figure 3. Threshold effect analysis revealed a notable inflection point at approximately 11 kg of gestational weight gain. Below this threshold, each additional 1 kg of weight gain was associated with a significantly reduced risk of PPD (OR = 0.905, 95% CI: 0.826–0.996, *P* = 0.036). Conversely, above 11 kg, each additional 1 kg of weight gain significantly increased the risk of PPD (OR = 1.059, 95% CI: 1.018–1.101, *P* = 0.005), as shown in Table 6.

**Table 6:** Threshold effect analysis of gain during pregnancy and PPD.

**Outcome**	**Adjusted OR (95% CI)^a^**	***p***
Threshold	11	
Gestational weight gain < 11 kg	0.905 (0.826, 0.996)	0.036
Gestational weight gain > 11 kg	1.059 (1.018, 1.101)	0.005
Logarithmic likelihood ratio test		0.009

^a^Adjusted for smoking, fetal sex, planned pregnancy, prenatal education classes, prenatal anxiety, perinatal sleep quality, marital relationship, and relationship with in-laws.

### 3.8 Importance ranking of influencing factors

**Figure 4:**
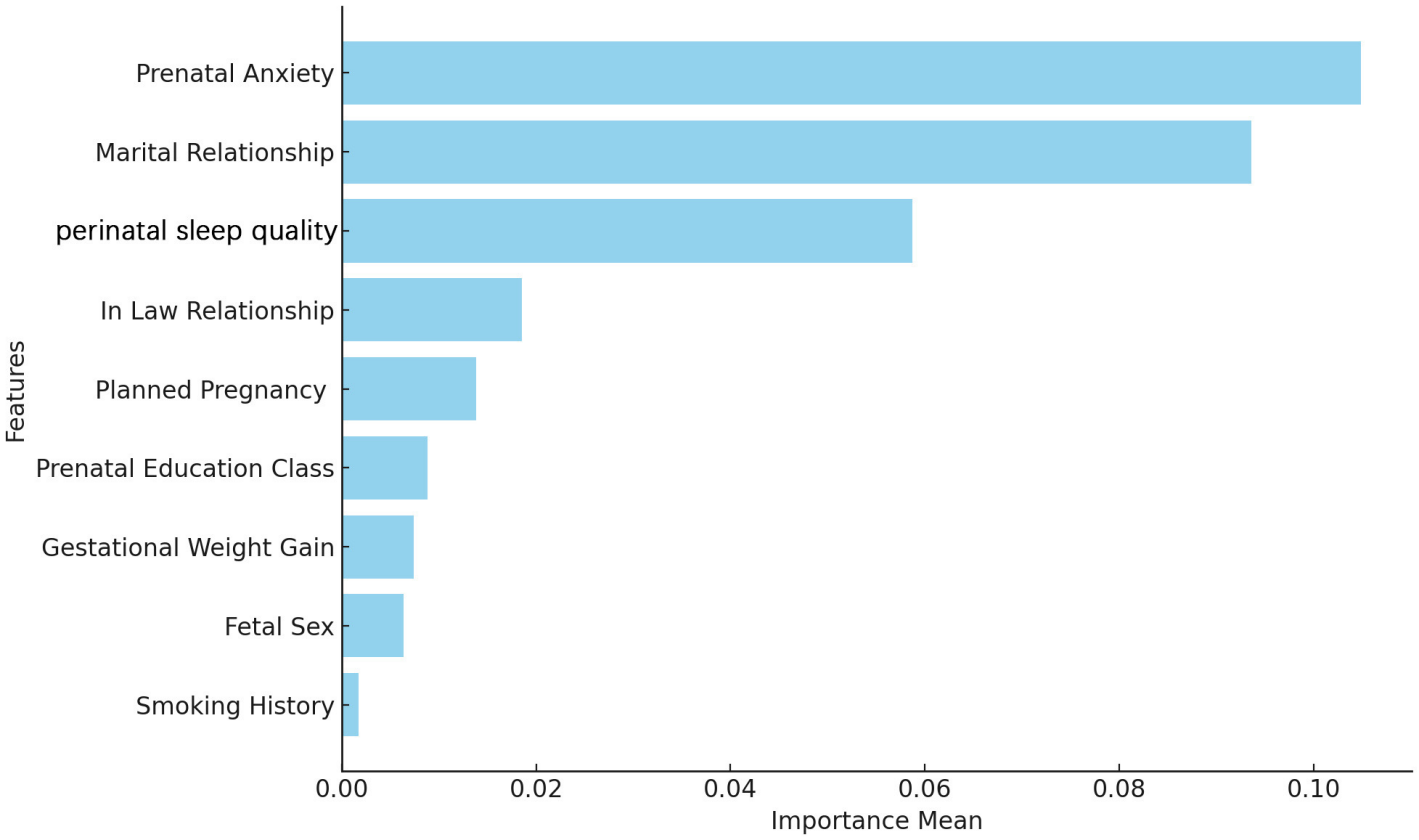
Feature importance ranking determined by random forest based on the ROC-AUC metric. Feature importance scores were estimated using a random forest algorithm, measured by the mean decrease in the area under the ROC curve (AUC).

Variables identified as statistically significant in the logistic regression analysis were analyzed using a random forest model to calculate importance scores based on the ROC-AUC metric. The ranking of variables by importance was as follows: prenatal anxiety, marital relationship, perinatal sleep quality, in-law relationship, planned pregnancy, prenatal education classes, gestational weight gain, fetal sex, and smoking history (Figure 4).

## 4 Discussion

### 4.1 High prevalence of PPD in Shenyang

PPD is a common health issue among postpartum women, posing significant threats to both maternal and neonatal health, and imposing substantial social and economic burdens. In this study, we utilized the widely used Edinburgh Postnatal EPDS to assess PPD, finding a positive screening rate of 23.57% (251/1,065) at 6 weeks postpartum. Compared to other domestic studies, our prevalence rate exceeds those reported in cross-sectional studies conducted in Shanghai (11.6%) and Shenzhen (11.8%) during routine postpartum check-ups (9, 14). Nationally, our reported detection rate also surpasses the PPD prevalence of 21.4% (95% CI: 15.2%−27.6%) identified in a meta-analysis of Chinese studies (3). Furthermore, our PPD detection rate is significantly higher than the global average of 14% (95% CI: 12.0%−15.0%) (3). Our findings also exceed the overall PPD prevalence of 13.6% (92/676) reported in a multinational study using the EPDS (15). This multinational study revealed significant differences in PPD prevalence across countries, with the highest rates observed in Ghana (26.0%, 13/50), followed by India (21.7%, 28/129) and Egypt (19.1%, 21/110), while Yemen, Iraq, and Syria reported lower rates of 8.5% (14/164), 7.7% (13/169), and 2.3% (1/43), respectively. In comparison, our study's prevalence is similar to the high rates in Ghana and India but markedly higher than those in Yemen, Iraq, and Syria.

These disparities may arise from multiple factors. First, geographic and socioeconomic differences could influence PPD incidence; for example, the advanced economic conditions in Shanghai and Shenzhen contrast with those in Shenyang. Second, variations in study design, such as our larger sample size (1,065 participants) compared to the multinational study (676 participants), may enhance detection rates. Third, differences in screening tools, timing, and diagnostic criteria likely contribute: this study used the EPDS at 6 weeks postpartum, whereas other studies may employ different instruments, time points, or cutoff scores, potentially affecting reported prevalence (8). Finally, cultural factors—such as societal attitudes toward mental health and access to support systems—may significantly influence prevalence across populations.

### 4.2 Predictors of PPD

Traditional approaches to analyzing factors influencing PPD often use univariate and multivariate analyses. While they can identify potential risk factors, they may struggle with complex, multidimensional data due to issues like multicollinearity and variable selection bias (16). As data mining advances and the complexity of medical records increases, conventional statistical methods face challenges in capturing hidden patterns and nonlinear relationships within high-dimensional data (17, 18). To address these limitations, this study utilized machine learning-based feature selection methods, such as LASSO regression and the Boruta algorithm. These techniques efficiently identify significant variables from complex data while reducing overfitting (19, 20), allowing for a more accurate identification of key factors linked to PPD. We identified statistically significant variables using multivariate logistic regression and enhanced interpretability by evaluating feature importance with a random forest model, ranking predictors based on changes in ROC-AUC. This approach clarifies which variables most impact PPD predictive performance, aiding in understanding potential influences. The variables were ranked by importance as follows: prenatal anxiety, marital relationship, sleep quality, in-law relationship, planned pregnancy, prenatal education classes, gestational weight gain, fetal sex, and smoking history.

#### 4.2.1 Prenatal anxiety

Prenatal anxiety is a well-established predictor of PPD (21). During pregnancy, women face significant physical, psychological, and hormonal changes, which can lead to increased anxiety or depression (22, 23). Postpartum, these psychological burdens may intensify due to physical exhaustion and childcare pressures, potentially progressing into PPD without adequate support and intervention. Research found that pregnant women with depression or anxiety symptoms had a notably higher prevalence of PPD compared to those without such symptoms (9). Rahman also highlighted that mothers experiencing antenatal depression face a significantly increased risk of depression within the first year postpartum (24). Consistent with these findings, this study also showed that antenatal anxiety was associated with a higher prevalence of PPD. This underscores the importance of early screening and timely intervention for antenatal psychological conditions, as well as providing targeted psychological support and counseling for pregnant women to prevent PPD.

#### 4.2.2 Poor marital relationships

Poor marital relationships have been identified as a significant risk factor for PPD (25). Postpartum hormonal fluctuations make mothers particularly emotionally vulnerable, underscoring the pivotal role of spousal support during this period (26). Spousal involvement not only mitigates psychological stress and stabilizes emotions but also fosters greater intimacy and trust between partners. Furthermore, it enhances mother-infant bonding by improving maternal confidence and the quality of parent-child interactions, thereby reducing the likelihood of PPD (27). In contrast, the absence of spousal support may exacerbate feelings of isolation and hinder psychological recovery (25, 27). Therefore, encouraging partner involvement and emotional support is essential for creating a supportive family environment for maternal wellbeing.

#### 4.2.3 Poor perinatal sleep quality

This study indicates a significant association between sleep quality and PPD. Pregnant women are more susceptible to sleep disorders and psychological distress due to physical, hormonal, and social changes. Inadequate sleep is strongly associated with postpartum fatigue and depression (28). Although the connection between sleep disturbances and PPD is well-established, the underlying mechanisms remain unclear. Some studies suggest poor sleep worsens PPD by reducing quality of life (29), while others view sleep disruption as a symptom of depression (30). Psychological stress may further exacerbate depressive symptoms by impairing sleep quality. Future research should clarify the causal pathways and explore targeted interventions. Timely interventions should be implemented to improve sleep quality in perinatal women experiencing poor sleep.

#### 4.2.4 Poor mother-in-law relationship

This study found that the relationship between mothers-in-law and postpartum women is closely linked to PPD risk, in line with existing research (11, 31). A positive relationship can offer emotional support, while conflict may increase psychological stress and elevate PPD risk (31). This is especially evident in Asian cultures, where co-residence with mothers-in-law can lead to tensions due to differing parenting philosophies (10). Future research should focus on how these relationships influence PPD, particularly regarding emotional support and role conflicts. Healthcare providers should assess family dynamics early and provide targeted support to reduce PPD risk. Improving mother-in-law and daughter-in-law relationships through family support and counseling can help reduce postpartum depression.

#### 4.2.5 Unplanned pregnancy

Unplanned pregnancies are a significant risk factor for PPD. A study found that women with unplanned pregnancies had higher rates of PPD compared to those with planned pregnancies (32), reflecting the psychological stress of being unprepared. Similarly, a meta-analysis of the Bangladesh population highlighted that unplanned pregnancies are linked to increased maternal depressive symptoms and adverse maternal and infant outcomes, further exacerbated by emotional distress, lack of support, and inadequate preparation (33). Therefore, pregnancy intention should be screened during routine prenatal care, and family planning as well as personalized mental health services should be integrated into primary care to provide tailored counseling and support for women with unintended pregnancies.

#### 4.2.6 Lack of attendance at prenatal education classes

Maternal education programs, such as prenatal classes, play a crucial role in reducing PPD risk. These programs enhance maternal knowledge of childbirth and parenting, alleviating anxiety and fear while improving mental health (3). By fostering social support and emotional management skills, they provide effective psychological interventions for postpartum women. Encouraging women to participate in prenatal education classes and providing mental health education and coping strategies can help them better adapt to pregnancy and the postpartum period.

#### 4.2.7 Gestational weight gain

This study, in line with a meta-analysis by Qiu (34), found a significant association between gestational weight gain and PPD risk. A nonlinear relationship was observed, with an inflection point at approximately 11 kg of gestational weight gain; weight associated with a reduced risk of PPD, while weight gain above it significantly increased the risk. The mechanisms behind this remain unclear, but one theory suggests that weight gain may influence PPD through the dysregulation of the hypothalamic-pituitary-adrenal (HPA) axis and inflammatory responses (35). Excessive weight gain, associated with obesity, could disrupt the HPA axis and trigger depression (36). Additionally, weight gain may activate inflammatory pathways linked to depression and impact the brain through insulin resistance, contributing to depressive symptoms (36, 37). Unhealthy weight gain can also affect body image and self-esteem, worsening mood disorders. Further research is needed to explore the underlying mechanisms. Healthcare providers should manage gestational weight gain through regular monitoring, nutritional guidance, and individualized counseling based on clinical recommendations to reduce the risk of PPD.

#### 4.2.8 High expectations for fetal sex

High expectations regarding fetal sex have been identified as a potential psychological stressor for expectant mothers. Cultural and familial pressures can amplify anxiety, particularly when the desired sex does not align with expectations. Such stress may contribute to emotional instability and elevate the risk of PPD (38). Therefore, for women with expectations regarding fetal sex, targeted follow-up and perinatal mental health education and support are recommended to reduce maternal stress and prevent PPD.

#### 4.2.9 Smoking history

Smoking is an independent risk factor for PPD, as confirmed by previous studies. A national cohort study involving 24,441 women showed that longer and greater smoking history before pregnancy increases PPD risk (39). Even women who quit smoking during pregnancy still faced heightened risk. This study emphasized that cumulative smoking history is a stronger predictor of PPD than smoking duration. Hence, smoking prevention should be integrated into PPD prevention strategies, with early interventions aimed at reducing smoking years to lower PPD risk. Additionally, for women with a history of smoking, closer follow-up and support should be provided.

### 4.3 Strategies for addressing PPD and future research directions

This study highlights the high prevalence of PPD in Shenyang, China, and identifies significant risk factors, offering crucial insights for healthcare professionals and policymakers. Firstly, given that prenatal anxiety, poor sleep quality, and strained marital relationships are major risk factors for PPD, it is recommended to enhance mental health support during the prenatal and postnatal periods. Healthcare institutions should implement routine screening and counseling to identify and support high-risk individuals, with specialized guidance for women experiencing prenatal anxiety and sleep disturbances. Secondly, the significant impact of family dynamics on PPD underscores the need for family-based mental health education to improve marital and in-law relationships. Although China has integrated perinatal depression screening into routine healthcare services, there is a need to strengthen implementation and coverage, and to enhance prenatal education programs to alleviate the psychological stress associated with unplanned pregnancies.

Future research should focus on the long-term effects of PPD, with longitudinal studies to observe its impact on maternal and infant health. Additionally, studies should consider cultural factors to develop adaptive interventions and explore the roles of fathers and partners in supporting postpartum mental health. By implementing these recommendations, healthcare providers and policymakers can more effectively identify and support individuals at high risk for PPD, thereby improving the mental health and overall wellbeing of postpartum women.

### 4.4 Strengths and limitations

This study offers several notable strengths. Conducted with a cohort of 1,065 postpartum women in Shenyang, it bolsters the statistical power and reliability of the findings. We examined an array of predictors and correlates of PPD using data from routine 42-day postpartum check-ups, which are readily identifiable and widely accessible across clinical settings. By integrating machine learning-based variable selection with logistic regression, we pinpointed key PPD predictors, effectively adjusted for potential confounders, and delivered robust statistical evidence. Furthermore, the application of RCS and threshold effect analysis clarified the association between gestational weight gain and PPD, providing actionable insights for targeted interventions.

This study has several limitations. Firstly, the EPDS is utilized as a screening tool rather than a diagnostic criterion, which may affect the accuracy of depression diagnosis. Additionally, this study employs a cross-sectional design, which only reveals associations between variables and the risk of PPD without establishing causality. Therefore, large-scale longitudinal studies are needed in the future to further validate these associations. Although this study includes a variety of clinical and demographic variables, it does not account for certain potential factors, such as a history of psychiatric disorders, which could significantly influence the results. Moreover, the study was conducted during the COVID-19 pandemic, a period when maternal mental health may have been impacted by pandemic-related stress, social isolation, and vaccination, yet these factors were not thoroughly assessed in this research. Furthermore, some variables, such as perinatal sleep patterns, were based on self-reported data from the participants rather than standardized assessments, potentially leading to information and measurement bias. The study was conducted in a hospital setting, which may limit the generalizability of the findings, and caution should be exercised when extrapolating the results to community or general populations. Lastly, the study focuses only on the postpartum period up to 6 weeks, lacking long-term follow-up, and thus does not explore the dynamic changes in depressive symptoms over time, warranting further investigation.

## 5 Conclusion

This study highlights a relatively high prevalence of PPD at 6 weeks postpartum among women in Shenyang, China. Key risk factors identified include antenatal anxiety, poor spousal relationships, low sleep quality, strained mother-in-law relationships, unintended pregnancies, lack of maternal education, excessive gestational weight gain, unrealistic expectations regarding fetal sex, and smoking. These findings emphasize the critical need for early identification of women at elevated risk of PPD and the implementation of targeted interventions addressing modifiable risk factors to effectively reduce its incidence.

## Data Availability

The data analyzed in this study is subject to the following licenses/restrictions: The datasets analyzed in current study are available from the corresponding author on reasonable request. Requests to access these datasets should be directed to syfykj2011@163.com.
