# Supplementary material for: Postpartum depression in Northeastern China: a cross-sectional study 6 weeks after giving birth
Source: Front Public Health. 2025 May 21;13:1570654. doi: 10.3389/fpubh.2025.1570654 (PMC12133524; doi:10.3389/fpubh.2025.1570654)
Supplement: Supplementary file 1 [file Table_1.docx]

**Detailed Definitions：**

**1.Smoking history**：according to the 2015 Global Adult Tobacco Survey questionnaire, if respondents answered “used to smoke but do not smoke now,” they were classified as former smokers. If respondents answered “never smoked,” they were classified as never smokers.

**2.Alcohol consumption history**： regardless of the type of alcohol, such as spirits, beer, wine, or rice wine, individuals who consumed alcohol at least once per week on average for a continuous or cumulative period of one year or more were classified as former drinkers.

**3**.**BMI** is calculated as weight (kg) divided by height squared (m²).

**4.Premature birth** is defined as a birth that occurs before 37 completed weeks of gestation.

**5.Adequacy of breast milk**：infant weight gain (at least 125 grams per week in the first 3 months), excretion patterns (6+ urinations and 2-5 golden-yellow, pasty stools daily), feeding behavior (regular swallowing sounds during breastfeeding, a satisfied expression afterward, and feeding intervals of 2-3 hours), and maternal breast changes (fullness before feeding, softening afterward, and occasional milk overflow).

**6**.**Pelvic floor muscle strength, encompassing both type I and type II muscle fibers**: was assessed through vaginal palpation using the Oxford grading system, which ranges from 0 to 5.Type I Muscle Fiber Strength: Type I fibers, also known as slow-twitch fibers, make up 70% of the total muscle fibers and are part of the deep muscle group. They are characterized by sustained contraction and endurance, providing support to the organs. Damage to these fibers can lead to vaginal wall laxity and pelvic organ prolapse. Type I muscle strength is categorized into six levels, from 0 to V, based on the duration a subject can sustain 40% of their maximum pelvic floor muscle contraction. A duration of 0 seconds is classified as level 0, 1 second as level I, 2 seconds as level II, and so on, with levels IV to V indicating normal muscle strength. Type II Muscle Fiber Strength: Type II fibers, or fast-twitch fibers, constitute 30% of muscle fibers and belong to the superficial muscle group. These fibers have greater explosive power and primarily function in controlling urination and defecation. Damage to these fibers can easily lead to stress urinary incontinence. Type II muscle strength is assessed by the number of times a subject can contract and relax the vaginal muscles at maximum force and speed, achieving over 60% of their maximum contraction strength. One repetition is classified as level I, two as level II, three as level III, and so on, with levels IV to V considered normal.

7.pelvic dynamic pressure (cmH2O):This refers to the pressure value recorded by a pressure balloon when the subject contracts their pelvic floor muscles with maximum force, with a normal range of 80–150 cmH_2_O. This value reflects the explosive strength of the pelvic floor muscle contraction, where 80 cmH_2_O represents a passing strength level, and 150 cmH_2_O indicates the maximum strength score.

Table 1: VIF Diagnostic Results

| **Variable Name** | **GVIF** | **Df** | **GVIF^(1/(2*Df))** |
| --- | --- | --- | --- |
| Smoke | 1.0522714071844 | 1 | 1.02580281106283 |
| Fetal_Sex | 2.10892088019498 | 1 | 1.45221240877324 |
| Planned_Pregnancy_ | 1.1270188189999 | 1 | 1.06161142561669 |
| Prenatal_Education_Class | 1.22594566072222 | 1 | 1.10722430461141 |
| Sleep_Status | 1.0654360844125 | 2 | 1.01597226044398 |
| Prenatal_Anxiety | 1.43309756679255 | 1 | 1.1971205314389 |
| Marital_Relationship | 1.66492183448139 | 1 | 1.29031850117767 |
| In_law_Relationship | 1.39690473485792 | 1 | 1.18190724460844 |
| Weight_Gain_During_Pregnancy | 1.0144668263344 | 1 | 1.00720743957459 |
| Feeding_Method | 1.04086820808335 | 1 | 1.02022948795031 |
